# Chronic Inhibition of Central Angiotensin-Converting Enzyme Ameliorates Colchicine-Induced Memory Impairment in Mice

**DOI:** 10.3797/scipharm.1203-06

**Published:** 2012-05-03

**Authors:** Himani Awasthi, Deep Kaushal, Hefazat Husain Siddiqui

**Affiliations:** 1 Amity University, Uttar Pradesh, India.; 2 Faculty of Pharmacy, Integral University, Lucknow, India.

**Keywords:** Perindopril, Renin angiotensin system, Acetylcholinesterase, Oxidative stress

## Abstract

Preclinical and clinical studies indicated involvement of the central renin-angiotensin system (RAS) in memory functions. However, the role of central angiotensin-converting enzyme (ACE) in memory function is still unclear. The present study investigated the involvement of central ACE in colchicine-induced memory impairment in the context of cholinergic function and oxidative stress. Memory impairment was induced by intracerebral colchicine administration in mice. The ACE inhibitor, perindopril (0.05 and 0.1 mg/kg/day), was administered orally for 14 days. Memory function was evaluated by the Morris water maze (MWM) test from the 14^th^ day on after colchicine injection. Donepezil was used as a standard. Parameters of oxidative stress and cholinergic function, ACE activity in serum and the brain were estimated after the completion of behavioral studies. Colchicine caused memory impairment as revealed by no significant change in latency to reach a hidden platform in the MWM test. Furthermore, there was a significant increase in MDA, ROS, and nitrite levels with a reduction in GSH level and acetylcholinesterase (AChE) activity in the brain of colchicine-treated mice. Colchicine significantly increased brain ACE activity without affecting serum ACE. Donepezil prevented colchicine-induced memory impairment in mice. The antidementic effect of perindopril may be attributed to reduced oxidative stress and improvement in cholinergic function. Moreover, the elevated brain ACE activity was also inhibited by perindopril. The study showed that central ACE plays an important role in colchicine-induced memory deficit, corroborating a number of studies that show that treatment with ACE inhibitors could be neuroprotective.

## Introduction

Drugs affecting renin angiotensin system (RAS), the angiotensin receptor (AT) blockers and angiotensin converting enzyme (ACE) inhibitors, have clinically been widely used as antihypertensive agents. Several studies showed that the brain has an intrinsic RAS which is independent of peripheral RAS and plays an important role in various physiological functions including cognition [1]. Clinical studies showed that administration of AT1 receptor blockers and ACE inhibitors in hypertensive patients improved cognitive function independent of their blood pressure lowering effects [2, 3]. Furthermore, postmortem analysis of AD brain showed an increase in ACE activity [4–6]. Clinical studies also suggested that ACE is involved in cognitive impairment in AD patients because ACE inhibitors delayed onset of dementia [7, 8] and significantly decreased the ACE activity in CSF [9].

A number of preclinical studies attempted to explore the role of ACE in memory function using experimental models of memory impairment [10, 11]. Recently it has been reported that chronic administration of ACE inhibitor ameliorated streptozotocin and scopolamine induced memory impairment by reducing cholinergic dysfunction and oxidative stress [12, 13]. It was also reported that ACE inhibition improved memory function in aged [14] and STZ induced diabetic rats [15]. Inhibition of ACE was also found to ameliorate cerebral hypoperfusion and amyloid beta induced memory impairment in rodents [16, 17].

Alzheimer’s disease (AD) is a neurodegenerative disorder that leads to deficits in learning and memory, altered personality, and ultimately death [1]. A large amount of evidence suggests that AD is associated with impairment in cholinergic system and elevated oxidative stress in brain [18, 19]. Intracerebroventricular administration of colchicine has been reported to impair memory function in rodents [20]. Colchicine is a microtubule disrupting agent which causes cytoskeletal alterations and dysfunction in axonal transport leading to death of basal forebrain cholinergic neurons causing cognitive impairment [21, 22]. It induces neurofibrillary degeneration by binding to tubulin and also causes excessive free radical generation and oxidative damage [23]. Colchicine-induced memory impairment is also associated with reduced endogenous antioxidant level and impaired cholinergic system in brain. Treatment with antioxidants and drugs improving cholinergic function has been reported to ameliorate colchicine-induced memory deficit [20, 21, 23].

Although previous studies reported beneficial effects of ACE inhibition in dementia using several models, its involvement in colchicine-induced oxidative stress, impaired cholinergic function and memory has not been investigated. Therefore, the present study explored the role of central ACE by utilizing intracerebral (IC) colchicine-induced model of memory impairment in mice. The clinically used ACE inhibitor, perindopril, was used as experimental pharmacological tool. The IC colchicine-induced dementia model was developed in mice and validated by using the clinically used acetylcholinesterase inhibitor, donepezil.

## Materials and methods

### Animals

Adult male Swiss albino mice (25–30 g) were purchased from the Laboratory Animal Services Division of Central Drug Research Institute, Lucknow. The animals were kept in polyacrylic cage (22.5 × 37.5 cm) and maintained under standard housing conditions (room temperature 24–27°C and humidity 60–65%) with a 12 h light and dark cycle. Food and water were available *ad libitum* but food was not allowed from 1 h prior to the behavioral study. All the experiments were carried out according to internationally followed ethical standards, and necessary approval from the Institutional Animal Ethics Committee was obtained (1213/ac/June 2008/CPCSEA).

### Materials

The biochemicals i.e. colchicine, chloral hydrate, sodium chloride (NaCl), sodium nitrite (NaNO_2_), sulphanilamide, napthaylamine diamine dihydrochloride, bovine serum albumin (BSA), acetylthiocholine iodide, 5,5′-dithiobis(2-nitrobenzoic acid) (DTNB), 1,1,3,3-tetra-ethoxypropane (TEP), glutathione (GSH), *N*-[3-(2-furyl)acryloyl]-l-phenylalanyl-glycylglycine (FAPGG), dichlorodihydrofluorescein diacetate (DCF-DA), and 2-thiobabituric acid (TBA) were purchased from Sigma-Aldrich, USA. Perindopril (Perigard) was purchased from local pharmacy store (Lucknow, India).

### Drug administration

#### Intracerebral (IC) administration of colchicine

The mice were anesthetized with chloral hydrate (300 mg/kg, IP). A midline sagittal incision was made in the scalp. A 27 gauge hypodermic needle attached to a 100 μl Hamilton syringe was inserted (2.5 mm depth) perpendicularly through the skull into the brain. Colchicine (1 and 3 μg/10 μl), dissolved in freshly prepared artificial CSF (aCSF), was administered slowly in a volume of 10 μl by intracerebral (IC) route. The site of injection was 2 mm from either side of the midline on a line drawn through the anterior base of the ears. The syringe was left in the place for an additional 2 min for proper diffusion of colchicine [24].

#### Experimental protocol and administration of perindopril

**Fig. 1:**
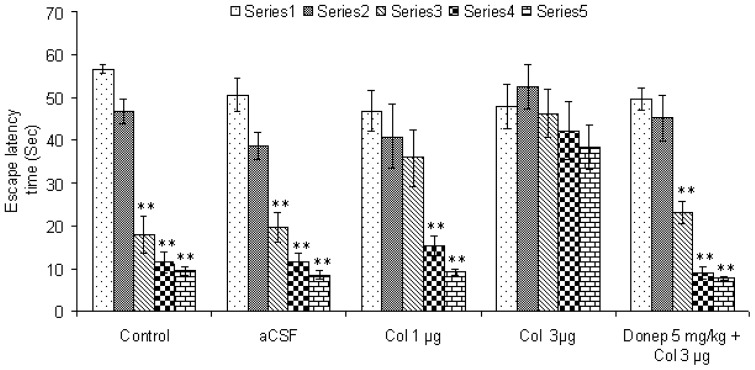
Standardization of colchicine-induced memory impairment model in mice. Results were expressed as mean latency time (sec) ± S.E.M. ^*^P < 0.05 and ^**^P < 0.05 in comparison to session 1.

Animals were randomly divided into eight groups of 5 mice each. For oral administration, perindopril was suspended in 1.0% w/v gum acacia just before administration in a constant volume of 10 ml/kg body weight. Non-hypotensive doses of perindopril, 0.05 mg/kg and 0.1 mg/kg, were administered orally daily for 14 days starting from 1 h before colchicine administration on 1^st^ day. The non-hypotensive doses of perindopril were selected based on previous studies [12, 13]. Control, aCSF and colchicine (1 and 3 μg) groups received vehicle of perindopril orally. Another group of mice was treated with perindopril 0.1 mg/kg to study *per se* effect on behavioral and biochemical parameters. Donepezil was administered at 5 mg/kg dose. (fig. 1)

### Evaluation of spatial memory by Morris Water Maze test

The Morris water maze consisted of a large circular pool (120 cm diameter and 50 cm height), filled with water (26 ± 2°C) to a depth of 30 cm. A black-colored round platform of 8 cm diameter was placed 1 cm below the surface of water in a constant position. The water was colored with non-toxic black dye to hide the location of the submerged platform.

On the 14^th^ day from colchicine injection, animals were subjected to Morris water maze test to evaluate memory function. Each mouse was given 3 trials per day for 5 consecutive days. The mice were given a maximum time of 60 s (cut-off time) to find the hidden platform and were allowed to stay on it for 30 s. Latency time to reach the platform was recorded in each trial. Mean latency time of all three trials is shown in the results. A significant decrease in latency time from that of 1^st^ session was considered successful learning. [24]

### Spontaneous locomotor activity

Locomotor activity was tested by Optovarimex activity meter 1 h before water maze trial on 14^th^ day after colchicine administration. Each group of mice was observed for 10 min taking readings every 2 min.

### Estimation of biochemical parameters

#### Tissue collection and preparation of homogenate

After the completion of behavioral studies, biochemical parameters were estimated on 18^th^ day after colchicine administration in mice brain excised under ether anesthesia after intra cardiac perfusion with chilled normal saline. A 10% (w/v) homogenate of brain samples (0.03 M sodium phosphate buffer, pH 7.4) was prepared by using homogenizer at a speed of 9,500 rpm.

#### Measurement of MDA

Malondialdehyde (MDA) level was estimated in mice brain as described previously [24]. Brain homogenate (300 μl) was mixed with 30% trichloroacetic acid (TCA), 5 N HCl followed by the addition of 2% thiobarbituric acid (TBA) in 0.5 N NaOH. The mixture was heated at 90°C for 15 min and centrifuged at 12,000 *g* for 10 min. The pink colour of the supernatant was measured at 532 nm using UV visible spectrophotometer. MDA concentration was calculated by using standard curve prepared with Tetra ethoxy propane and expressed as nmol/mg protein.

#### Measurement of GSH

Glutathione (GSH) level was estimated by Ellman method [25]. The brain homogenate and 10% TCA (1:1) were mixed and kept on ice for 10 min, then centrifuged at 2,000 *g* for 10 min at 4°C and supernatant was collected and used for GSH estimation. The supernatant was mixed with phosphate buffer (pH 8.4) and DTNB. The absorbance was read at 412 nm using UV visible spectrophotometer. GSH concentration was calculated by using standard curve prepared with reduced glutathione and expressed as μg/mg protein.

#### Nitrite estimation

Nitrite was estimated in the mice brain using the Griess reagent [12]. Equal volume of Griess reagent and processed tissue sample was mixed, and absorbance was measured at 542 nm using UV visible spectrophotometer. Nitrite concentration was calculated using a standard curve for sodium nitrite and expressed in μg/mg protein.

#### Measurement of reactive oxygen species (ROS) by spectrofluorometry

Amount of ROS in brain was measured using 2,7-dichlorofluorescin (DCF)-diacetate, which gets converted into highly fluorescent DCF. In brief, brain was homogenized in ice-cold 40 mM Tris-HCl buffer (pH 7.4). The resulting brain homogenate was incubated with DCF-DA (5 μM) for 30 min in a 37°C water bath. The formation of the fluorescent product DCF was monitored by fluorescence spectrometer with excitation wavelength of 488 nm and emission wavelength of 530 nm [25].

#### Sample preparation and assay of AChE activity

The brain homogenate was mixed with equal volume of 1% Triton X-100 and centrifuged at 100,000 *g* at 4°C for 60 min. Supernatant was collected and stored at 4°C for acetylcholinesterase (AChE) estimation by using DTNB and acetylthiocholine iodide. The enzyme activity was measured by UV visible spectrophotometer at 412 nm with an interval of 15 s. The specific activity of AChE is expressed in μmoles/min/mg protein [12, 25].

#### Estimation of angiotensin converting enzyme (ACE) activity

Angiotensin converting enzyme (ACE) activity was measured by a synthetic substrate, N-[3-(2-furyl)acryloyl]-l-phenylalanyl-glycylglycine (FAPGG). The reaction was monitored at 340 nm. Serum ACE activity was estimated by adding 200 μl of the substrate solution to 10 μl of serum. The reaction was monitored at 340 nm taking readings up to 30 min at an interval of 5 min using UV visible spectrophotometer. Serum ACE activity was expressed as unit/liter. The brain homogenate was mixed with equal volume of 1% Triton X-100 and centrifuged at 100,000 *g* at 4°C for 60 min. Supernatant was collected and stored at 4°C for estimation of ACE activity. To estimate ACE activity 10 μl of this supernatant was mixed with 200 μl of substrate solution and reaction was monitored at 340 nm at 37°C for 30 min in UV visible spectrophotometer. Brain ACE activity was expressed in Units/mg protein [12].

#### Protein estimation

Protein was measured in brain samples by using Folin reagent as described previously [12, 25]. Bovine serum albumin (BSA) (1 mg/ml) was used as standard and measured in the range of 0.01–0.1 mg/ml.

#### Statistical analysis

The results are expressed as mean ± S.E.M. Statistical analysis of Morris water maze and biochemical values was done by one way ANOVA followed by Tukey’s test.

## Results

### Effect of perindopril on colchicine-induced memory impairment in mice

As shown in fig. 1 control [F (4, 20) = 66.48, P < 0.01] and aCSF [F (4, 20) = 38.3, P < 0.01] treated mice showed significant decrease in escape latency time (ELT) from session three onward indicating spatial learning. To induce memory impairment, colchicine was administered intracerebrally at 1 and 3 μg/mice dose. Memory function was evaluated from 14^th^ day after colchicine administration.

Colchicine at 1 μg dose [F (4, 20) = 10.63, P < 0.05] showed significant decrease in ELT at session 4 and 5 while the dose of 3 μg caused memory deficit as shown by no significant decrease [F (4, 20) = 0.93, P > 0.05] in ELT throughout all the sessions. Furthermore, the colchicine-induced memory deficit model was standardized by using clinically used antidementic drug donepezil. Administration of donepezil (5 mg/kg, PO) for 14 days attenuated colchicine-induced memory impairment as shown by significant decrease [F (4, 20) = 42.53, P < 0.01] in latency time from session 3 onward.

**Fig. 2:**
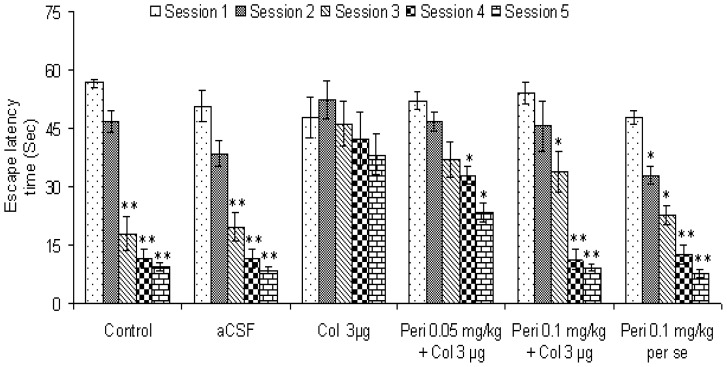
Effect of perindopril on colchicine-induced memory impairment in mice. Results were expressed as mean latency time (sec) ± S.E.M. ^*^P < 0.05 and ^**^P < 0.05 in comparison to session 1.

To study the role of angiotensin converting enzyme (ACE) in colchicine-induced memory impairment, the ACE inhibitor perindopril was used as an experimental pharmacological tool. Perindopril was administered at 0.05 and 0.1 mg/kg dose for 14 days starting from colchicine injection. As shown in fig. 2, perindopril dose dependently reversed colchicine-induced memory impairment in mice. Perindopril 0.05 mg/kg [F (4, 20) = 13.0, P < 0.01] treated mice showed significant decrease in ELT from session 4 onward, whereas higher dose [F (4, 20) = 23.79, P < 0.01] decreased ELT from session 3 onward. Moreover, perindopril *per se* treatment enhanced spatial memory as there was a significant decrease [F (4, 20) = 64.8, P < 0.01] in ELT from session 2 onward in comparison to session 1. However, the mean latency to reach platform during session 1 did not differ significantly [F (7, 16) = 1.13, P > 0.05] among different groups.

### Biochemical estimations

#### Effect of perindopril on serum and brain ACE activity in colchicine-induced memory deficit mice

**Fig. 3:**
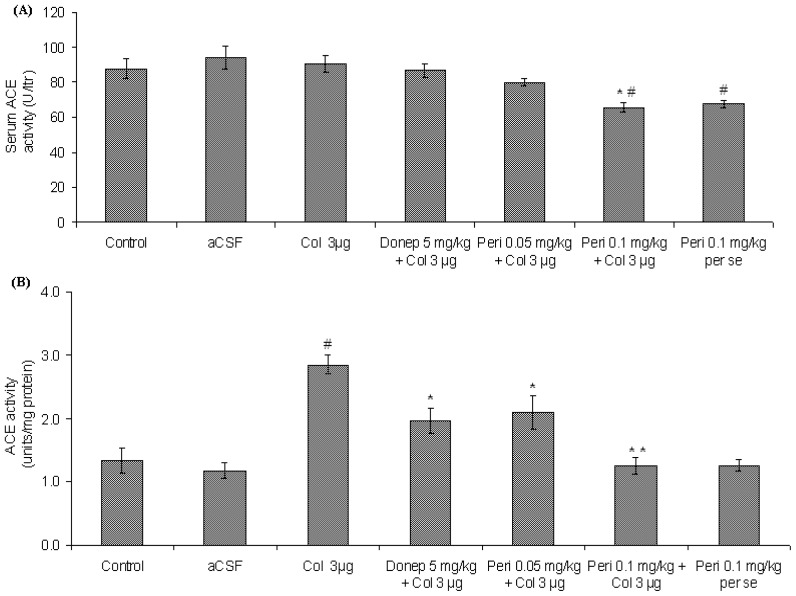
a: Effect of perindopril pretreatment on serum ACE activity in colchicine (IC) induced memory deficit mice. Serum ACE activity was expressed as mean Units/liter ± S.E.M. ^*^P<0.05 in comparison to control and vehicle groups and ^#^P<0.05 in comparison to colchicine group. b: Effect of perindopril pretreatment on brain ACE activity in colchicine (IC) induced memory deficit mice. Brain ACE activity was expressed as mean Units/mg protein ± S.E.M. ^#^Significant increase (^#^P<0.05) in comparison to control group and ^*^P<0.05 in comparison to colchicine (IC) group.

As shown in fig 3a, IC administration of colchicine had no significant effect on serum ACE activity in comparison to that of control and aCSF groups [F (2, 12) = 2.19, P>0.05]. Furthermore, administration of donepezil did not affect serum ACE activity significantly (P>0.05). However, perindopril dose dependently decreased serum ACE activity [F (4, 20) = 9.84, P<0.05] in comparison to control, vehicle and colchicine treated mice.

There was a significant increase [F (2, 12) = 14.14, P<0.01] in brain ACE activity following IC colchicine administration when compared with control and vehicle groups. Preventive administration of perindopril significantly decreased [F (2, 12) = 11.95, P<0.01] ACE activity in brain in comparison to colchicine group. Furthermore, donepezil significantly ameliorated (P<0.05) colchicine induced elevation in brain ACE activity (Fig. 3b).

#### Effect of perindopril on MDA level in colchicine-induced memory deficit mice brain

**Fig. 4:**
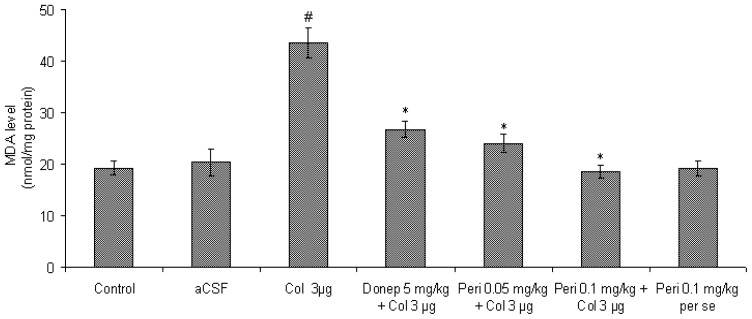
Effect of perindopril on MDA (nmol/mg protein) level in mice brain. Results were expressed as mean ± S.E.M. ^#^Significant difference (^#^P<0.01) vs control and aCSF group and ^*^P < 0.01 vs colchicine group.

Intracerebral administration of colchicine caused a significant increase in MDA level in mice brain as compared with control and aCSF groups [F (2, 12) = 33.30, P<0.01]. However, administration of aCSF (IC) had no significant (P>0.05) effect on MDA level as compared to control (Fig. 4). The standard antidementic drug donepezil attenuated colchicine-induced lipid peroxidation as shown by a significant reduction (P<0.05) in MDA level in mice brain.

Preventive treatment with perindopril (0.05 and 0.1 mg/kg, PO) doses dependently decreased MDA level [F (2, 12) = 38.96, P<0.01] in colchicine-injected mice brain. However, perindopril *per se* (0.1 mg/kg, PO) had no significant [F (2, 12) = 0.14, P>0.05] effect on MDA level in comparison to control and vehicle group.

#### Effect of perindopril on GSH level in colchicine-induced memory deficit mice brain

A significant fall in the levels of GSH was observed in the colchicine group as compared to the control and aCSF treated groups [F (2, 12) = 24.86, P<0.01]. The clinically used antidementic drug donepezil significantly increased (P<0.05) GSH level in colchicine injected mice brain.

**Fig. 5:**
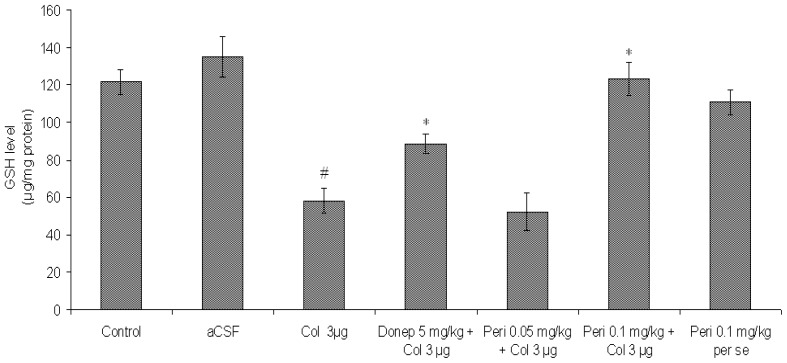
Effect of perindopril on GSH (μg/mg protein) level in mice brain. Results were expressed as mean ± S.E.M. ^#^Significant difference (^#^P<0.01) vs control and aCSF group and ^*^Significant difference (^*^P < 0.05 and **P<0.01) vs colchicine group.

Treatment with perindopril dose dependently prevented the decrease in GSH levels in the brain of colchicine-injected mice [F (2, 12) = 21.59, P<0.01]. However, perindopril *per se* (0.1 mg/kg, PO) had no significant [F (2, 12) = 2.09, P>0.05] effect on GSH level in comparison to control and vehicle group (Fig. 5).

#### Effect of perindopril on nitrite level in colchicine-induced memory deficit mice brain

**Fig. 6:**
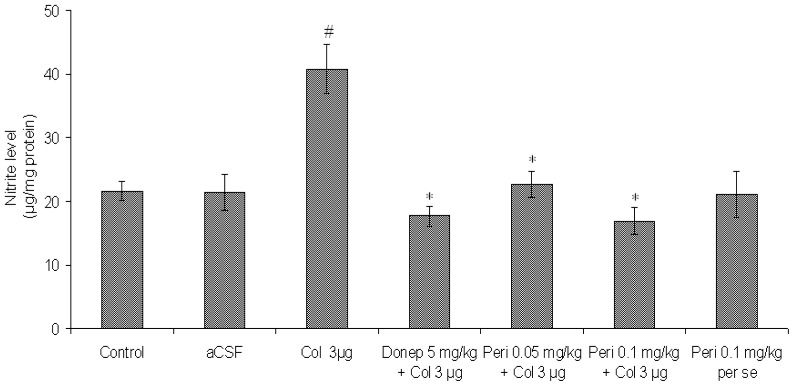
Effect of perindopril on nitrite (μg/mg protein) level in mice brain. Results were expressed as mean ± S.E.M. ^#^Significant difference (^#^P<0.01) vs control and aCSF group and ^*^P<0.01 vs colchicine group.

A significant rise in nitrite level was observed in the brain of colchicine treated mice in comparison to control and aCSF groups [F (2, 12) = 13.57, P<0.01]. Perindopril treatment significantly inhibited this increase in nitrite levels in colchicine treated mice [F (2, 12) = 19.85, P<0.01] (Fig. 6). Donepezil also caused a significant decrease (P<0.05) in nitrite level in comparison to colchicine group.

#### Effect of perindopril on ROS level in colchicine-induced memory deficit mice brain

**Fig. 7:**
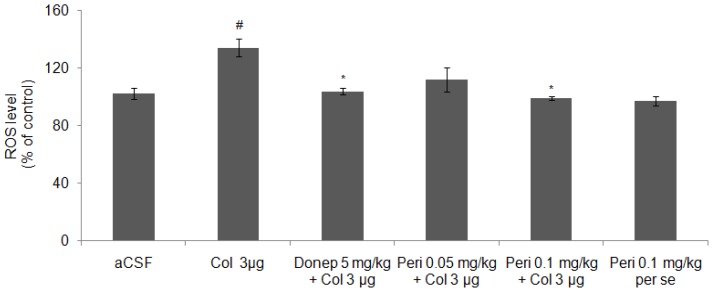
Effect of perindopril on ROS (% of control) level in mice brain. Results were expressed as mean ± S.E.M. ^#^Significant difference (^#^P<0.01) vs control and aCSF group and *P<0.01 vs colchicine group.

As shown in fig. 7, IC administration of colchicine caused a significant increase [F (2, 12) = 15.26, P<0.01] in ROS level in comparison to control and vehicle groups. Preventive treatment with donepezil significantly (P<0.05) reduced ROS level in comparison to colchicine group.

Furthermore, perindopril dose dependently prevented colchicine-induced generation of ROS in mice brain. There was a significant reduction in ROS level in perindopril 0.1 mg/kg [F (2, 12) = 8.96, P<0.05] treated group but lower dose failed to prevent colchicine-induced ROS generation in mice brain. However, perindopril per se had no significant effect on ROS generation [F (2, 12) =1.13, P>0.05] in comparison to control and vehicle groups.

#### Effect of perindopril on AChE activity in colchicine-induced memory deficit mice brain

**Fig. 8:**
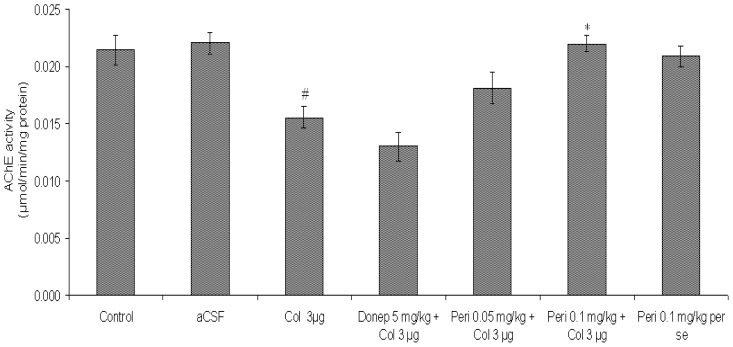
Effect of perindopril on AChE activity (μg/min/mg protein) in mice brain. Results were expressed as mean ± S.E.M. ^#^Significant difference (^#^P<0.01) vs control and aCSF group and *P<0.01 vs colchicine group.

AChE activity was significantly decreased in the colchicine treated mice brain when compared with control and aCSF groups [F (2, 12) = 11.11, P<0.01]. Furthermore, donepezil also decreased AChE activity in colchicine injected mice. Chronic treatment with perindopril significantly [F (2, 15) = 9.69, P<0.01] prevented the decrease in AChE activity in colchicine treated group (Fig. 8).

## Discussion

The present study showed that angiotensin converting enzyme (ACE) plays a crucial role in memory deficit induced by intracerebral (IC) colchicine because treatment with perindopril, an ACE inhibitor, prevented memory impairment, oxidative stress and cholinergic dysfunction in mice.

Colchicine is a cytotoxic agent which binds irreversibly to microtubules and causes their depolymerization, thereby inhibiting their assembly. Microtubules are vital components of the neuronal cytoskeleton and play a crucial role in cell growth and differentiation, axonal and dendritic transport. It has been reported that central administration of colchicine induce memory impairment in rodents by causing cholinergic neurodegeneration and oxidative stress [21, 22]. In the present study intracerebral administration of colchicine at a dose of 3 μg/mice induced spatial memory impairment as indicated by no significant reduction in escape latency time in Morris water maze test. However, lower dose of colchicine failed to induce memory deficit. Therefore, further studies were carried out by using colchicine at 3 μg/mice dose. This finding is in agreement with previous studies reporting impairment in memory following colchicine administration [20–23]. Furthermore, the colchicine-induced memory impairment model was validated by clinically used antidementic-anticholinesterase drug donepezil. Preventive treatment with donepezil for 14 days ameliorated colchicine-induced memory impairment in mice. Kumar et al. (2007) [21] also reported that chronic administration of acetylcholinesterase (AChE) inhibitor rivastigmine prevented colchicine-induced dementia in rats.

To study the involvement of central ACE in colchicine-induced memory impairment, an ACE inhibitor perindopril was used as an experimental pharmacological tool. Perindopril was administered chronically for 14 days in colchicine injected mice and memory function was tested by Morris water maze. Perindopril dose dependently ameliorated colchicine-induced dementia in mice implicating central ACE in memory function. Furthermore, *per se* treatment of perindopril improved memory function as shown by significantly reduced retention latencies as compared to control animals. There was no significant effect on locomotor activity excluding the possibility that alteration in locomotor activity may have contributed to the observed behavioral effects. The cognitive effects of ACE inhibitors have previously been investigated in various models of memory deficit [12–17]. However, to the best of our knowledge, this is the first study reporting antidementic effect of ACE inhibitor.

Clinical studies have shown an elevated ACE activity in various brain regions of Alzheimer’s disease (AD) patients [4]. Furthermore, it has been reported that memory impairment induced by streptozotocin (STZ) or amyloid beta was associated with increased ACE activity in brain [12, 16]. In the present study, we also had similar findings as colchicine significantly increased ACE activity in mice brain without affecting serum ACE activity indicating involvement of central ACE in memory deficit. Administration of perindopril decreased ACE activity in serum and brain. Moreover, perindopril *per se* treatment significantly decreased ACE activity in brain. The exact mechanism of ACE activation is not known but it may be due to elevated oxidative stress by colchicine. Recently, it has been reported that STZ induced oxidative stress leads to upregulation of central ACE activity and mRNA expression in rat brain regions [12]. Additionally, Usui et al. [26] showed an elevated serum ACE activity in rats treated with nitric oxide synthase inhibitor N(omega)-nitro-l-arginine methyl ester and this effect was prevented by an antioxidant drug, N-acetylcysteine [26]. These observations suggest that oxidative stress plays an important role in the regulation of serum and brain ACE activity.

In agreement with previous reports [20–22], we found elevated nitrosative (increased nitrite level) and oxidative stress (decreased GSH and, increased MDA and ROS) in colchicine treated mice brain. This enhancement in oxidative stress markers may be due to increased formation of Ang II, due to increased ACE activity, which stimulates NADPH oxidase that plays a pivotal role in the development of oxidative stress by producing superoxides [12]. ACE inhibition by perindopril showed anti oxidative action as evidenced by reduced MDA and elevated GSH levels. Furthermore, perindopril pretreatment reduced the superoxide formation in mice brain. ACE inhibition, by decreasing Ang II, limits the stimulation of vascular NADPH oxidase, thereby preventing the increased superoxide formation associated with activation of the RAS. Pretreatment with perindopril decreased nitrite level also.

Central cholinergic system plays an important role in memory formation and retrieval. The neurotransmitter acetylcholine is degraded by the enzyme AChE. Therefore, the use of AChE inhibitors is the most effective pharmacological approach for the symptomatic treatment of AD. Colchicine has been reported to cause destruction of hippocampal granule cells and septohippocampal pathways with a reduction in AChE activity [27, 28]. Also, in the present study cholinergic system was affected in colchicine-induced memory deficit, as there was a significant reduction in AChE activity in mice brain. Perindopril normalized the decreased AChE activity in IC colchicine treated mice. However, perindopril *per se* had no significant effect on AChE activity in brain.

## Conclusion

The present study showed that treatment with perindopril ameliorated colchicine-induced memory impairment in mice implicating central ACE in memory function. Furthermore, the beneficial effects of ACE inhibition may be attributed to reduced oxidative stress and cholinergic dysfunction in the brain. This study corroborated a number of clinical findings that show that inhibition of central ACE could be neuroprotective.
